# First person – Yuka Kobayashi

**DOI:** 10.1242/dmm.049369

**Published:** 2021-11-24

## Abstract

First Person is a series of interviews with the first authors of a selection of papers published in Disease Models & Mechanisms, helping early-career researchers promote themselves alongside their papers. Yuka Kobayashi is first author on ‘ Early manifestations and differential gene expression associated with photoreceptor degeneration in *Prom1*-deficient retina’, published in DMM. Yuka is assistant professor in the lab of Prof. Kazuhiro Kimura at Yamaguchi University Graduate School of Medicine, Ube, Japan, investigating ophthalmology.


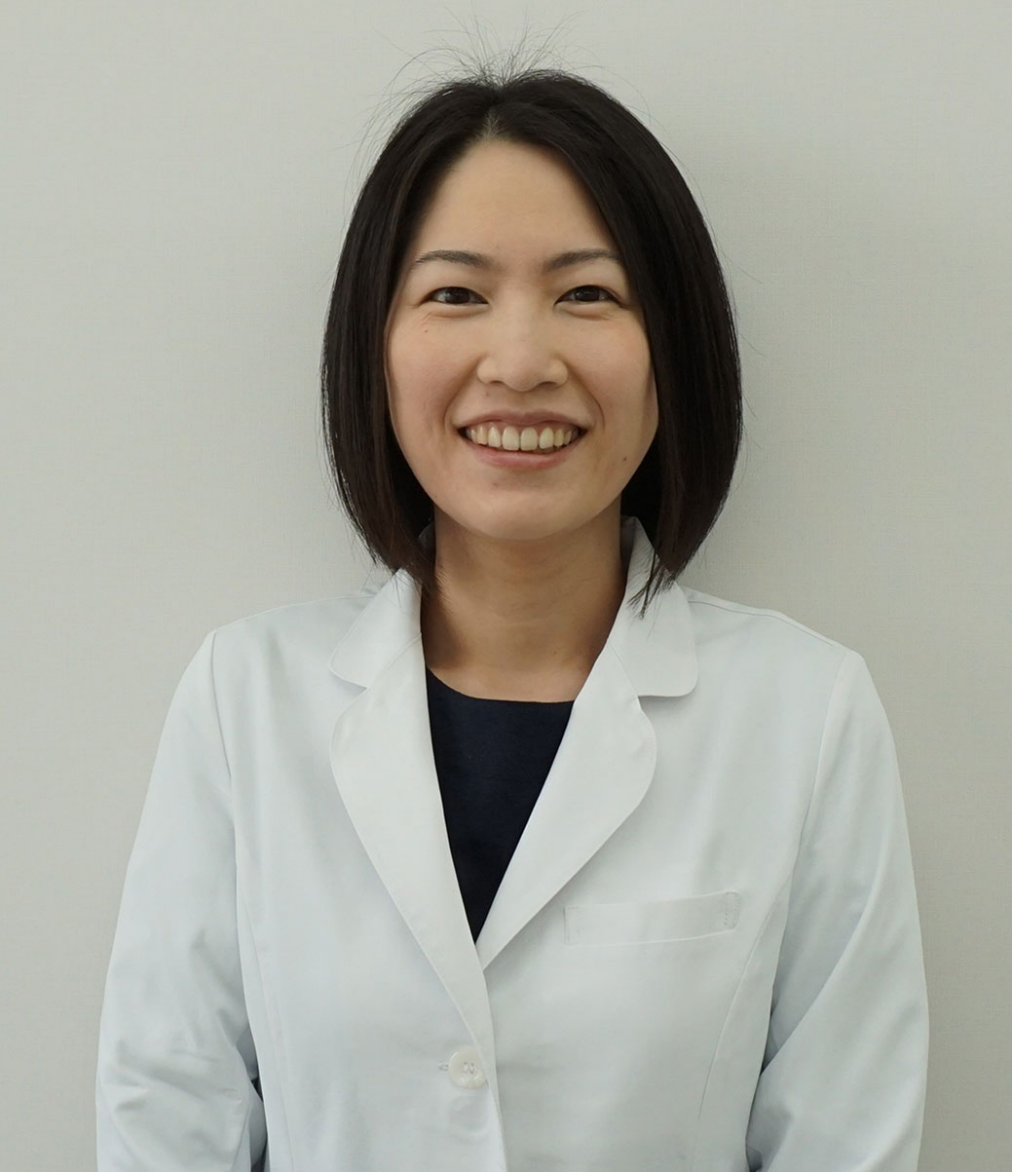


**Yuka Kobayashi**

**How would you explain the main findings of your paper to non-scientific family and friends?**

Seeing is fundamental to our lives, and the loss of this ability has a major impact on our quality of life. Retinitis pigmentosa has one of the most severe impacts among ocular diseases, and half of all cases are known to be hereditary. It is estimated that more than 5 million people worldwide suffer from retinitis pigmentosa, but there are currently no therapeutic methods. Recently, some cutting-edge treatments, such as regenerative medicine, have been developed, but their versatility is still low, and standard treatments, including medication, are awaited. Since mutations in prominin-1 (*Prom1*) are also known to cause hereditary retinitis pigmentosa, we started to investigate the primary effects of mutations in this gene, particularly signalling and gene expression, and attempted to find drugs to compensate them. Using the Prom1-mutant mice, we found that mutations in this gene had no effect on the development of retinal tissue, but that continuous light stimulation altered the expression of a number of genes, resulting in the necrosis of photoreceptor cells in the retina. In particular, endothelin-related genes were found to cause apoptosis, and drugs that blocked their signalling system were administered to partially block photoreceptor cell apoptosis.

“Seeing is fundamental to our lives, and the loss of this ability has a major impact on our quality of life.”

**What are the potential implications of these results for your field of research?**

Our results provide insights into the primary cell biological conditions that cause retinitis pigmentosa and contribute to the development of a treatment for this disease. In particular, by targeting the initial state of the disease, we expect to be able to delay the disease progression and provide effective treatment.

**What are the main advantages and drawbacks of the model system you have used as it relates to the disease you are investigating?**

Because there are differences in the size and structure of the retina between mice and humans, deliberative clinical trials are needed to ensure that treatments identified in mice can be applied to humans. In addition, as there are more than 60 causative genes for retinitis pigmentosa, there is a concern that a single mouse model may not be sufficient to find a general treatment.

**What has surprised you the most while conducting your research?**

It was surprising to find that the retinal disintegration occurred rapidly due to the photoreceptor cell death within a short period of time, while there were no signs at all soon after birth.

**Figure:**
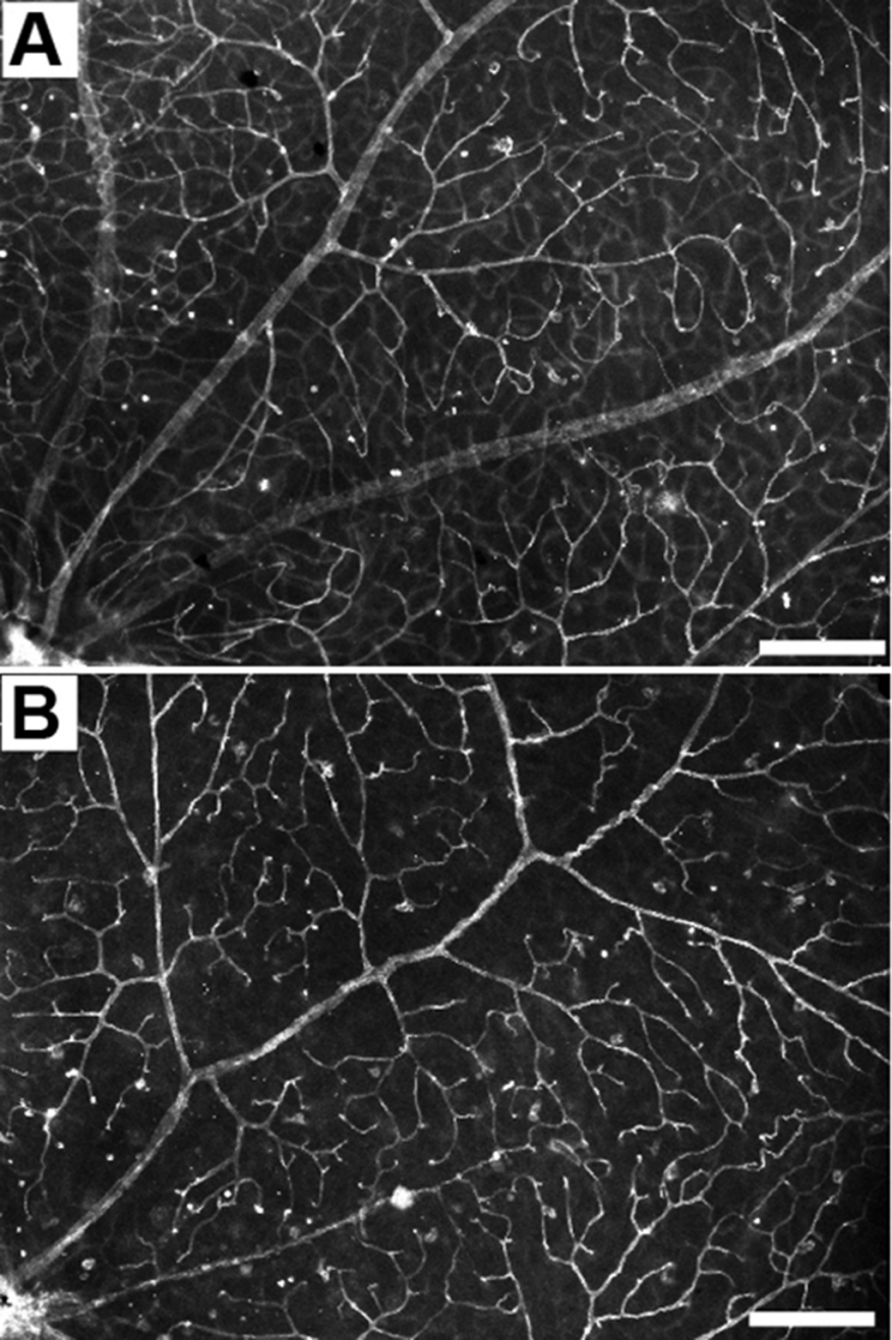
**Images of healthy retina (A) and narrowed vessels found in the *Prom1*-knockout retina (B).** Scale bars: 100µm.

**Describe what you think is the most significant challenge impacting your research at this time and how will this be addressed over the next 10 years?**

In this study, we have identified signalling pathways and potential therapeutic agents that may be effective in retinitis pigmentosa. Hopefully, detailed gene expression profiles during the disease progression will be elucidated at a single-cell level in the future and further effective drugs and/or their combinations will be elucidated.

“[…] it is important to have opportunity for early-career scientists to freely challenge research themes of their own design to keep them motivated.”

**What changes do you think could improve the professional lives of early-career scientists?**

I believe to improve the environment for early-career scientists, so that they can concentrate on their research, such as financial support in the form of grants or salaries, the enhancement of research equipment and facilities, and the creation of a system to improve their skills. Moreover, it is important to have opportunity for early-career scientists to freely challenge research themes of their own design to keep them motivated.

**What's next for you?**

I would like to be involved in understanding the molecular mechanisms of retinitis pigmentosa and other eye diseases caused by other genes. As a clinician myself, my next goal is to deepen both basic and clinical research and to develop therapies based on solid evidence from molecular and cellular biology.
